# Supplementary figures and images for: Transcriptomic analysis reveals candidate genes for male sterility in Prunus sibirica
Source: PeerJ. 2021 Oct 20;9:e12349. doi: 10.7717/peerj.12349 (PMC8541319; doi:10.7717/peerj.12349)

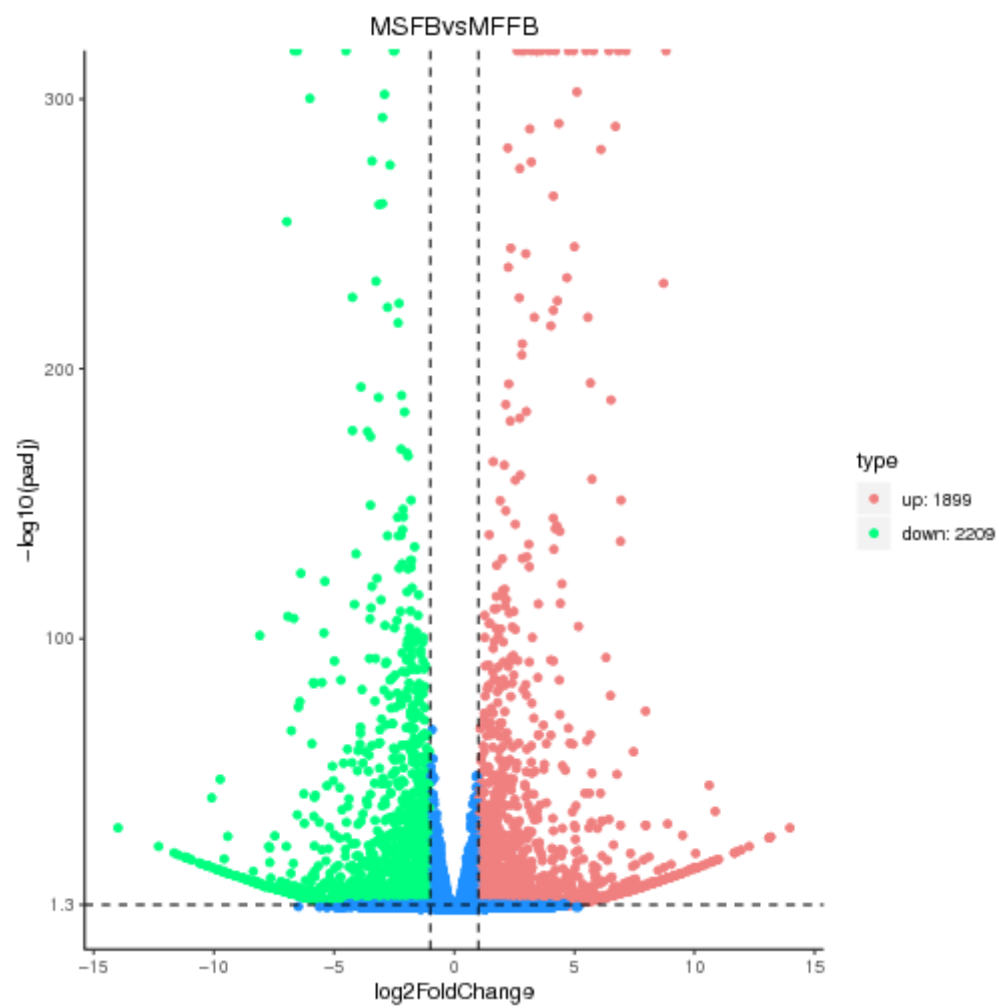

Supplement: Supplemental Information 1 [file peerj-09-12349-s001.pdf]

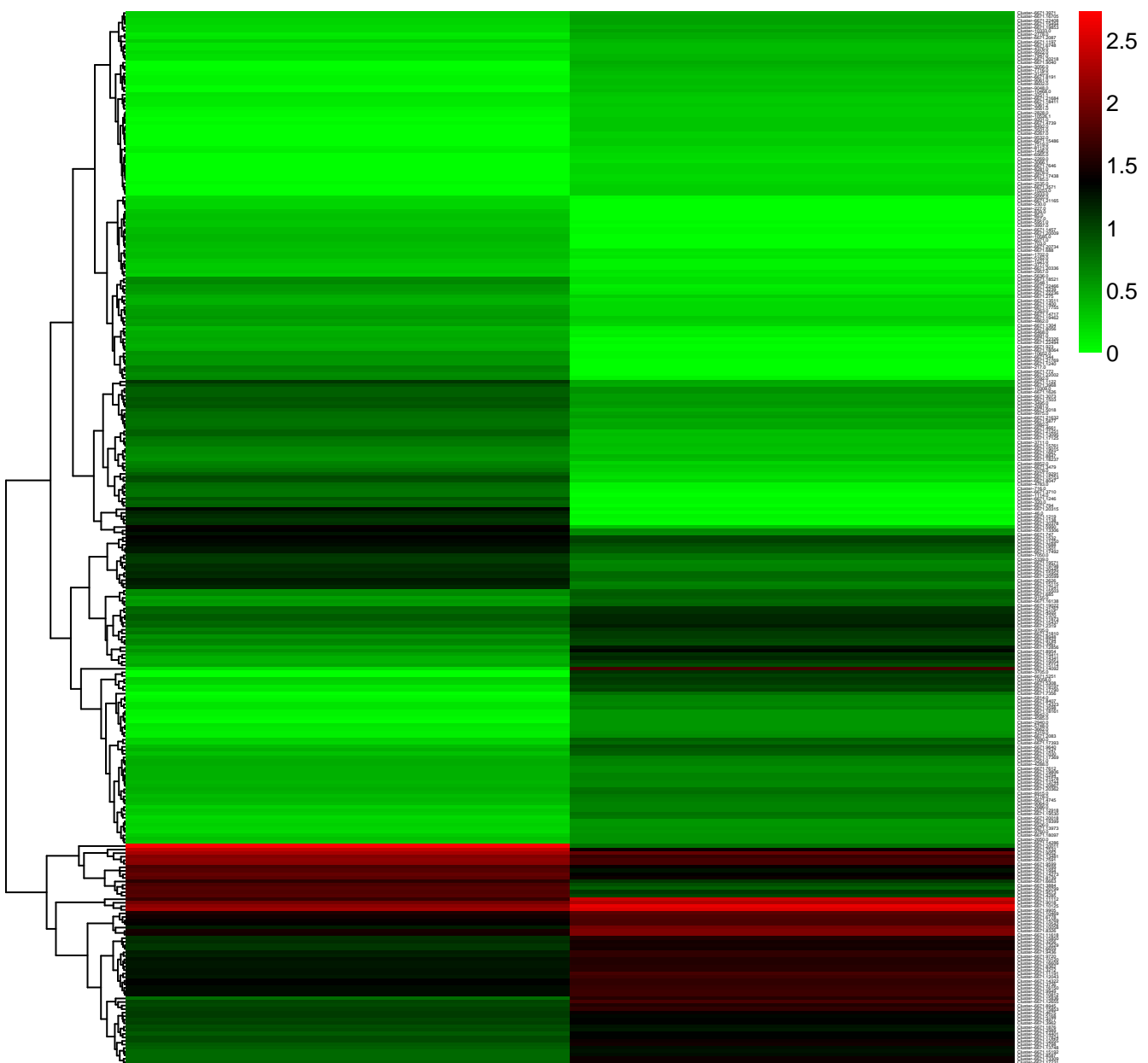

MSFB

MFFB

Supplement: Supplemental Information 2 [file peerj-09-12349-s002.pdf]

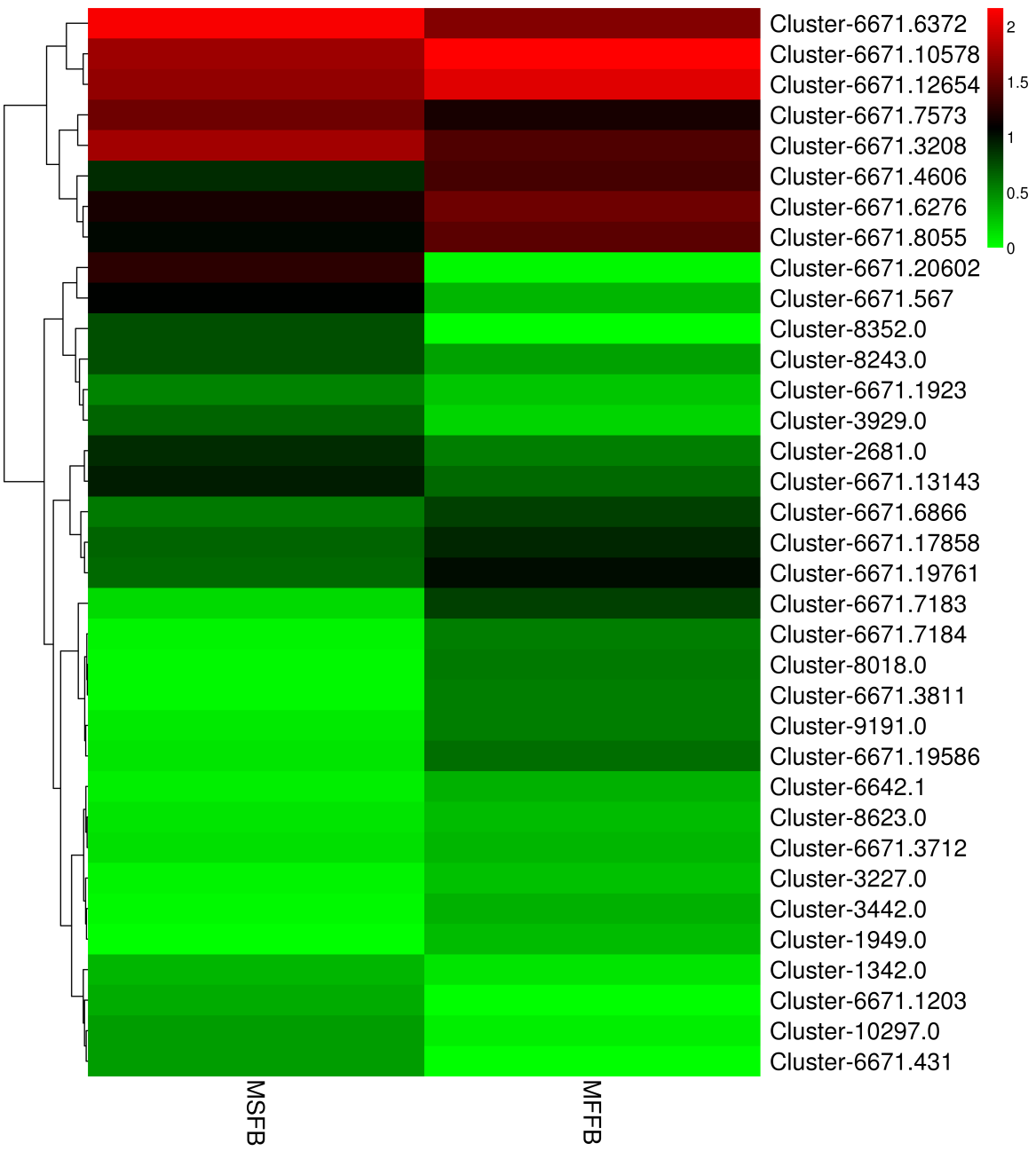

Supplement: Supplemental Information 3 [file peerj-09-12349-s003.png]
