# Supplementary material for: Transcriptomic analysis reveals candidate genes for male sterility in Prunus sibirica
Source: PeerJ. 2021 Oct 20;9:e12349. doi: 10.7717/peerj.12349 (PMC8541319; doi:10.7717/peerj.12349)
Supplement: Supplemental Information 4 [file peerj-09-12349-s004.docx]

**Table S1 Sequence information of primers for RT-qPCR**

| **Gene ID** | **Primer** | **Primer sequence（5’-3’）** |
| --- | --- | --- |
| *Cluster-10585.0* | *Cluster-10585.0-F*  *Cluster-10585.0-R* | ACCACAAGGGCAATCCACAAC  TTTCAGGGAGATTTACTGGGGATT |
| *Cluster-6671.20011* | *Cluster-6671.20011-F*  *Cluster-6671.20011-R* | TCCATCACTGCCAACAAAACTT  CAGAACCAGACCAAACCAACC |
| *Cluster-6671.6372* | *Cluster-6671.6372-F*  *Cluster-6671.6372-R* | GAGGAAAACTCCACTCTTAGCC  GTTCTATGCCCTCAAGTATCGC |
| *Cluster-6671.3208* | *Cluster-6671.3208-F*  *Cluster-6671.3208-R* | GCATCAGTACCCCCTAATAACTTCC  TGCCACCCATTCATTCATAACG |
| *Cluster-2681.0* | *Cluster-2681.0-F*  *Cluster-2681.0-R* | AGAAGGGAATCAAAGCCATCG  CTCGGTTTGTAGCCTTGGATTG |
| *Cluster-6671.20378* | *Cluster-6671.20378-F*  *Cluster-6671.20378R-* | ATTATGCTGCGGAGGTTACTGCT  TGGCTCACTAGGTATCACATCGACTAG |
| *Cluster-257* | *Cluster-257-F*  *Cluster-257-R* | TCATAACCAACAAAATAGTTCCGC  GATGGAATGGTTTGAGCAGTGT |
| *Cluster-6671.1138* | *Cluster-6671.1138-F*  *Cluster-6671.1138-R* | CAAATCTATGGGTTTCTGGGGTC  AGTGGTGTCTATGTCTAGTGCCGAG |
| *Cluster-6671.20009* | *Cluster-6671.20009-F*  *Cluster-6671.20009-R* | CTCATTGTGGACTCAGATGGTGT  ACACTTTTCCAGTCTCACCTTCAT |
| *Cluster-2940.0* | *Cluster-2940.0-F*  *Cluster-2940.0-R* | CAACGGAACCCTGGATAGTAAGAC  CCTCGTTACCACCTCCGTCTC |
| *Cluster-2269.0* | *Cluster-2269.0-F*  *Cluster-2269.0-R* | CCTCAACAATGTCACGGCAGAA  GCAGCCCGAACGCATACAAC |
| *Cluster-4585.0* | *Cluster-4585.0-F*  *Cluster-4585.0-R* | GTTCCATTTTTGTCATTGTTGCC  AGAGGGTGGGTTGTGGTCGT |
| *Cluster-8492.0* | *Cluster-8492.0-F*  *Cluster-8492.0-R* | AAATGAAAAAGGCGGTGACTC  TGGGCAAAGATTGTTGTGGT |
| *Cluster-9048.0* | *Cluster-9048.0-F*  *Cluster-9048.0-R* | TATGGACGCATCTACAAGGACG  TGAGGGATGTTCTGTATCGGTTG |
| *18SrRNA* | *18SrRNA-F*  *18SrRNA-R* | AAACGGCTACCACATCCA  CACCAGACTTGCCCTCCA |
