# Supplementary material for: Transcriptomic analysis reveals candidate genes for male sterility in Prunus sibirica
Source: PeerJ. 2021 Oct 20;9:e12349. doi: 10.7717/peerj.12349 (PMC8541319; doi:10.7717/peerj.12349)
Supplement: Supplemental Information 5 [file peerj-09-12349-s005.docx]

**Table S2** **Quantitative examination results of total RNA**

| **Sample** | **Concentration/ng.μl^-1^** | ***OD*_260/280_** | ***OD*_260/230_** | **RIN value** | **25S/18S** |
| --- | --- | --- | --- | --- | --- |
| MSFB_1 | 647 | 2.24 | 2.10 | 9.70 | 1.5 |
| MSFB_2 | 670 | 2.24 | 2.11 | 9.70 | 1.5 |
| MSFB_3 | 619 | 1.74 | 2.18 | 9.60 | 1.4 |
| MFFB_1 | 541 | 2.55 | 0.73 | 9.70 | 1.5 |
| MFFB_2 | 474 | 2.34 | 0.66 | 9.90 | 1.6 |
| MFFB_3 | 452 | 2.26 | 0.76 | 9.90 | 1.6 |
