# Supplementary material for: Transcriptomic analysis reveals candidate genes for male sterility in Prunus sibirica
Source: PeerJ. 2021 Oct 20;9:e12349. doi: 10.7717/peerj.12349 (PMC8541319; doi:10.7717/peerj.12349)
Supplement: Supplemental Information 6 — Q30: The percentage of bases with Phred value > 20 to the total bases, where Phred =−10Log10e. [file peerj-09-12349-s006.docx]

**Table S3 Quality statistics for transcriptome sequencing (RNA-seq) data**

| **Summary** | **MSFB_1** | **MSFB_2** | **MSFB_3** | **MFFB_1** | **MFFB_2** | **MFFB_3** |
| --- | --- | --- | --- | --- | --- | --- |
| Total raw reads | 40,662,496 | 44,220,630 | 47,693,148 | 60,453,772 | 63,078,522 | 55,062,658 |
| Total clean reads | 40,030,500 | 43,401,986 | 46,806,738 | 59,289,208 | 61,826,716 | 54,034,224 |
| Clean reads rate (%) | 98.45 | 98.15 | 98.14 | 98.07 | 98.02 | 98.13 |
| Clean bases (Gb) | 6.00 | 6.51 | 7.02 | 8.89 | 9.27 | 8.11 |
| Error rate (%) | 0.02 | 0.02 | 0.03 | 0.02 | 0.02 | 0.02 |
| Q30 percentage (%) | 94.30 | 94.22 | 94.10 | 94.74 | 94.98 | 94.82 |
| GC content (%) | 45.67 | 45.65 | 45.67 | 45.85 | 45.88 | 45.85 |
| Total mapped reads | 32,466,930 | 35,354,396 | 38,083,930 | 48,239,700 | 50,665,794 | 44,238,190 |
| Total mapping reads percentage (%) | 81.11 | 81.46 | 81.36 | 81.36 | 81.95 | 81.87 |

Q30: The percentage of bases with Phred value > 20 to the total bases, where Phred=-10Log_10_e.
