# Supplementary material for: Transcriptomic analysis reveals candidate genes for male sterility in Prunus sibirica
Source: PeerJ. 2021 Oct 20;9:e12349. doi: 10.7717/peerj.12349 (PMC8541319; doi:10.7717/peerj.12349)
Supplement: Supplemental Information 7 [file peerj-09-12349-s007.docx]

**Table S4 The summary statistics of the assembled transcripts and unigenes**

|  | Min length(bp) | Median length(bp) | Max length(bp) | Average length(bp) | N50(bp) |
| --- | --- | --- | --- | --- | --- |
| Transcript | 301 | 1586 | 16978 | 1885 | 2633 |
| Unigene | 301 | 1044 | 16978 | 1568 | 2520 |
