# Supplementary material for: Transcriptomic analysis reveals candidate genes for male sterility in Prunus sibirica
Source: PeerJ. 2021 Oct 20;9:e12349. doi: 10.7717/peerj.12349 (PMC8541319; doi:10.7717/peerj.12349)
Supplement: Supplemental Information 8 [file peerj-09-12349-s008.docx]

**Table S5 The success rate of functional annotation in transcriptome unigenes**

| **Databases** | **Numbers of the annotated unigenes** | **Percentage (%)** |
| --- | --- | --- |
| Nr | 20,510 | 59.66 |
| Nt | 26,505 | 77.10 |
| KEGG | 8,244 | 23.98 |
| SwissProt | 18,688 | 54.36 |
| Pfam | 18,025 | 52.43 |
| GO | 18,025 | 52.43 |
| KOG | 5,875 | 17.08 |
| All Database | 3,283 | 9.54 |
| At least one Databse | 27,798 | 80.86 |
| Total Unigenes | 34,377 | 100.00 |
